# Assessment of Individualized Mean Perfusion Pressure Targets for the Prevention of Cardiac Surgery-Associated Acute Kidney Injury—The PrevHemAKI Randomized Controlled Trial

**DOI:** 10.3390/jcm12247746

**Published:** 2023-12-18

**Authors:** Alicia Molina-Andujar, José Rios, Gaston J. Piñeiro, Elena Sandoval, Cristina Ibañez, Eduard Quintana, Purificación Matute, Rut Andrea, Teresa Lopez-Sobrino, Jordi Mercadal, Enric Reverter, Irene Rovira, Ana Maria Villar, Sara Fernandez, Manel Castellà, Esteban Poch

**Affiliations:** 1 Nephrology and Kidney Transplantation Department, Hospital Clinic, 08036 Barcelona, Spain; amolinaa@clinic.cat (A.M.-A.); gjpineir@clinic.cat (G.J.P.); 2 Department of Clinical Farmacology, Hospital Clinic and Medical Statistics Core Facility, 08036 Barcelona, Spain; jrios@clinic.cat; 3 Institut d’investigacions Biomèdiques Agustí Pi i Sunyer (IDIBAPS), 08036 Barcelona, Spain; randrea@clinic.cat (R.A.); telopez@clinic.cat (T.L.-S.); 4 Faculty of Medicine, Universitat Autònoma de Barcelona, 08193 Barcelona, Spain; 5 Cardiovascular Surgery Department, Hospital Clinic, 08036 Barcelona, Spain; esandova@clinic.cat (E.S.); equintan@clinic.cat (E.Q.); mcaste@clinic.cat (M.C.); 6 Anesthesiology Department, Hospital Clínic, 08036 Barcelona, Spain; cribanez@clinic.cat (C.I.); pmatute@clinic.cat (P.M.); mercadal@clinic.cat (J.M.); irovira@clinic.cat (I.R.); 7 Cardiology Department, Hospital Clínic, 08036 Barcelona, Spain; 8 Liver and Digestive ICU, Liver Unit, Hospital Clínic, 08036 Barcelona, Spain; ereverte@clinic.cat; 9 Perfusion Department, Hospital Clínic, 08036 Barcelona, Spain; villar@clinic.cat; 10 Medical Intensive Care Unit, Hospital Clínic, 08036 Barcelona, Spain; sfernanm@clinic.cat

**Keywords:** acute kidney injury, cardiac surgery, mean perfusion pressure, prevention, clinical trial

## Abstract

Background: Retrospective studies support that mean perfusion pressure (MPP) deficit in cardiac surgery patients is associated with a higher incidence of acute kidney injury (CS-AKI). The aim of our study was to apply an algorithm based on MPP in the postoperative period to determine whether management with an individualized target reduces the incidence of CS-AKI. Methods: Randomized controlled trial of patients undergoing cardiac surgery with extracorporeal circulation. Adult patients submitted to valve replacement and/or bypass surgery with a high risk of CS-AKI evaluated by a Leicester score >30 were randomized to follow a target MPP of >75% of the calculated baseline or a standard hemodynamic management during the first postoperative 24 h. Results: Ninety-eight patients with an eGFR of 54 mL/min were included. There were no differences in MAP and MPP in the first 24 h between the randomized groups, although a higher use of noradrenaline was found in the intervention arm (38.78 vs. 63.27, *p* = 0.026). The percentage of time with MPP < 75% of measured baseline was similar in both groups (10 vs. 12.7%, *p* = 0.811). MAP during surgery was higher in the intervention group (73 vs. 77 mmHg, *p* = 0.008). The global incidence of CS-AKI was 36.7%, being 38.6% in the intervention group and 34.6% in the control group (*p* = 0.40). There were no differences in extrarenal complications between groups as well. Conclusion: An individualized hemodynamic management based on MPP compared to standard treatment in cardiac surgery patients was safe but did not reduce the incidence of CS-AKI in our study.

## 1. Introduction

Post-operative acute kidney injury (AKI) is due to multiple factors, among which there are hemodynamic changes, both ischemia and congestion, that compromise renal perfusion. In cardiac surgery, cardiopulmonary bypass is added as an element that contributes to AKI by various mechanisms such as hemolysis, changes in medullar perfusion or increased systemic inflammatory response. The incidence of AKI in patients undergoing cardiac surgery can be up to 35% and 2–5% require renal replacement therapy (RRT) during the episode of AKI. If AKI is present after cardiac surgery, the risk of death during admission increases five-fold and if RRT is required, mortality can be up to 50% [1].

The KDIGO 2012 guidelines proposed a package of actions for the prevention of AKI [2]. Among the most important measures we find the close monitoring of creatinine and diuresis and the optimization of hemodynamic parameters and volaemia. For the latter, it is recommended to consider dynamic or functional monitoring based on algorithms. In that regard, a randomized controlled trial of post-operative cardiac surgery patients at risk of AKI showed that the application of these measures significantly reduced the development of moderate or severe AKI [3].

The optimal blood pressure target in the critically ill patient is controversial. Asfar and colleagues randomized critically ill septic patients to either a standard target mean arterial pressure (MAP) of 65–70 mmHg (standard) or an intensive MAP of 75–80 mmHg. There were no differences in terms of AKI, mortality and need for RRT except patients with a previous history of hypertension, where those assigned to a target MAP of 75–80 mmHg required less RRT but without significant differences in mortality [4].

In recent years, renal congestion has been studied as a contributor to the development of AKI [5], since the two components involved in renal mean perfusion pressure (MAP-central venous pressure [CVP]) have proven its importance. Retrospective studies in post-cardiac surgery and septic patients support that a mean perfusion pressure (MPP) deficit >20–25% (i.e., the difference between basal or preoperative MPP and postsurgery MPP) is associated with a higher incidence of AKI [6,7,8]. Data from these studies are collected from patient arrival in the intensive care unit (ICU), but intraoperative perfusion pressure deficit may already be contributing to AKI. Historically, MAP during extracorporeal circulation surgery is maintained at 50–70 mmHg because of the risk of the presumed increased risk of hemorrhagic complications with higher levels. Recent studies have shown that maintaining MAP between 80–100 mmHg is not associated with greater cerebral/cardiac complications [9]. On the contrary, regarding renal function, it has been demonstrated that MAP < 60 mmHg during cardiac surgery is associated with a higher incidence of AKI [10].

Thus, prospective studies are needed to support the importance of maintaining a reduced perfusion pressure deficit in order to extend algorithms in intensive care units that may take this parameter into account in case it proves a reduction in the AKI incidence.

The aim or our study is to apply an algorithm that includes calculated baseline MPP using standard monitoring tools to determine whether individualized hemodynamic management based on MPP during cardiac surgery and in the first 24 h of the postoperative management in the ICU compared to the standard one reduces the incidence of post-operative cardiac surgery AKI (CS-AKI). 

## 2. Methods

From 1 October 2019 to 20 September 2022, one thousand and twenty-two patients who underwent cardiac surgery in Hospital Clínic de Barcelona were initially screened to participate in the nonblinded randomized controlled trial (ClinicalTrials.gov Identifier: NCT04005105, https://classic.clinicaltrials.gov/ct2/history/NCT04005105 (accessed on 3 March 2023)). Inclusion criteria were use of cardiopulmonary bypass (CPB) during surgery, valve replacement and/or coronary artery bypass graft (CABG) with or without additional procedures including myomectomy, maze or left atrial appendage closure, score >30 in Leicester score and age ≥ 18 years. Exclusion criteria were chronic kidney disease stage V, kidney transplant recipients, renal immune diseases (glomerulonephritis/vasculitis etc.), endocarditis, history of AKI 7 days before surgery, use of ventricular assist devices or vasoactive drugs, use of intravenous vasodilator drugs, constrictive pericarditis, emergent surgeries or need for targeted brain perfusion therapy. Patients that participated in other interventional studies were also excluded. 

Leicester score (LS) includes age, sex, body mass index (BMI), smoking habits, dyspnea, diabetes, peripheral vascular disease, hypertension, preoperative hemoglobin, preoperative estimated glomerular filtration rate (e GFR), time from catheterism to surgery, presence of triple vessel disease, ejection fraction, emergency surgery, and type of surgery [11]. Our group described that both discrimination and calibration for AKI were better defined when the LS was used compared to the Cleveland Clinic Score and Euroscore II in a Spanish cohort [12].

Patients meeting the inclusion criteria without any exclusion criteria were asked to sign informed consent once they were admitted to the hospital for surgery. Hospital admissions were scheduled between 15 and 22 h before surgery. After signing informed consent, patients were randomized to standard of care (SoC) or intensive treatment by block randomization. The person in charge of enrollment and randomization was the principal investigator of the study.

The Institutional Ethics Committee approved the study protocol (HCB/2019/0501; 12 June 2019).

### 2.1. Protocol Description

Once randomized, MAP was measured three times before surgery and the mean between those was considered the patient basal MAP. Once in surgery, when the patient was intubated but before incision, basal CVP was measured during apnea. Basal MPP was considered the difference between basal MAP and CVP. Cardiac index was measured with a pulmonary artery catheter (Edwards Lifesciences Swan-Ganz^®^, Washington, DC, USA) in all included patients. 

For the Intervention group, the intraoperative protocol consisted of target MAP > 75% of basal MAP or >60 mmHg if the value was <60 mmHg. We consider that CVP during extracorporeal circulation is around 0, so this parameter was not included during surgery. In the ICU Protocol, once the patient was in ICU physicians were asked to follow an algorithm that included MPP. New goal compared to SoC protocol was MPP > 75% of basal value during 24 h. Also, initiation of furosemide was requested if repeated CVP > 14 when not contraindicated.

For the SoC group, in the intraoperative protocol the target MAP was >60 mmHg. In the ICU protocol, physicians were asked to follow standard of care protocols based on maintaining cardiac index > 2.2 l/min/m^2^ and MAP > 65 mmHg. Furosemide was initiated based on physician criteria.

The primary outcome was AKI during the first week after cardiac surgery. AKI was defined based on KDIGO guidelines: increase in serum creatinine by ≥0.3 mg/dL (≥26.5 µmol/L) within 48 h or increase in serum creatinine ≥ 1.5 times baseline within 7 days in the intention to treat (ITT) population defined as all randomized patients. 

### 2.2. Variables

Baseline variables were recorded before surgery from the preanesthetic report and medical interview. Those variables included: date of surgery, gender, smoking habit, weight, body mass index (BMI), laboratory values (hemoglobin, creatinine, eGFR by CKD-EPI formula), type of surgery, New York Heart Association classification (NYHA), use of diuretics, Angiotensin-converting enzyme inhibitor (ACEi)/ Angiotensin II receptor blockers (ARB), history of previous AKI, Charlson index, Leicester score, Euroscore, history of previous cardiac surgery, number of antihypertensive drugs, left ventricle ejection fraction, triple vessel disease. Baseline MAP, CVP and MPP were obtained as described in the protocol description.

Intraoperative variables were obtained both from perfusionist and anesthesiologists reports, lab results and hemodynamic monitors. Variables included: surgical time aaortic cross-clamping time, extracorporeal time, lactate and hematocrit values, volume administered, diuretic use, use of vasoactive drugs and dose, transfusion need, perfusion flow and MAP measured every 1–5 min according to availability.

Variables during admission were obtained from electronic health reports. Variables included: daily creatinine values until discharge, MAP and MPP during the first 24 h (MAP measured by continuous invasive blood pressure monitoring and CVP measured every 60–120 min), diuresis and balance during the first 24 h, vasoactive drug use and duration, transfusion need, date of ICU discharge and date of hospital discharge.

### 2.3. Statistical Analysis

In this open-label clinical trial, we employed a randomized (1:1), controlled design to investigate the efficacy and safety of the follow target of MPP >75% baseline vs. SoC follow-up during the first 24 h with diagnosis of AKI within the first week after surgery as the primary endpoint. 

The trial protocol assumed that in the SoC group the proportion of AKI would be 30% and that it was possible to reduce it to 15%, thus requiring 120 patients per group for a two-sided type I error of 5% and a power of 80%.

During 2020, a state of alarm was declared due to the epidemic caused by COVID-19, which meant that many clinical trials and new patient enrolments had to be halted. In 2021, normality returned to the participating center for the inclusion and follow-up of patients in clinical trials, and so the inclusion of patients in the current trial was resumed as well. In September 2022, the decision was made to stop enrolment due to low recruitment and administrative reasons.

Finally, 98 patients were available for analysis. Variables have been described by median and interquartic range [IQR: 25th; 75th percentiles] or absolute and relative frequencies (%), for quantitative and qualitative variables, analyzing differences between groups by Mann–Whitney U test or Fisher’s exact test respectively.

The estimation of relative risk of AKI, within the first week between groups, was done by estimating the Odds Ratio (OR) and its 95% confidence interval (CI95%) using a logistic regression model. Since it was not possible to include all expected patients, as a robustness analysis, the CI95% of the OR was also estimated using a resampling bootstrap method with *n* = 1000 replicates. 

All analyses were performed with a bilateral type I error of 5% which, except in the case of the analysis of the main variable, should be considered nominal with no inferential value. The statistical package SPSS VER 26 (Armonk, NY, USA: IBM Corp) was used for all analyses.

## 3. Results

**Figure 1:**
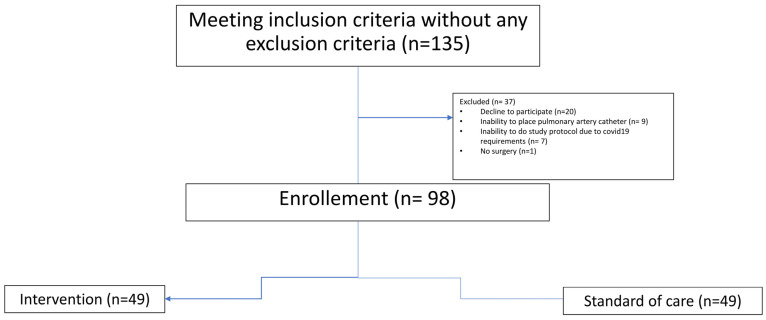
Flow chart of subject selection and randomization.

During the study period, one hundred and thirty-five patients met all inclusion criteria without any exclusion criteria. A total of 37 patients were excluded: 20 declined to participate; 9 patients had contraindication for use of pulmonary artery catheter; 7 patients could not follow the protocol due to COVID-19 requirements and in one patient surgery was not performed during the study period after anesthesiologist re-examination. Finally, ninety-eight patients were included in the study and were randomized into the intervention group (forty-nine) or SoC group (forty nine) Figure 1.

### 3.1. Cohort Baseline Characteristics

Baseline characteristics of both groups are depicted in Table 1. Median age was 74.78 years [IQR: 67.24; 78.78] and 82.65% were male. Hypertension was present in 84 patients (85.71%), with a greater prevalence in bypass surgery patients (82%) followed by combined surgeries (86%). As for renal function, median serum creatinine levels were 1.27 mg/dL with median eGFR of 54 mL/min. Median Leicester score was 42.80 [IQR: 35.30; 51.80]. Valve substitution was the most frequent intervention (37.76%) and 14.29% of the patients had two or more valves replaced. The valve that was most frequently replaced was aortic (64.79%) followed by mitral (12.68%). As for combined valve replacement, the most frequent valve combination was aortic and mitral (50% of combined valve procedures). Eighteen patients needed an extra procedure associated with the CABG or valve replacement (either myomectomy, maze procedure or left atrial appendage closure).

### 3.2. Hemodynamic Variables and Fluid Balance during Intervention Period

There were no statistical differences in surgical time, aortic cross-clamping time and CPB time in both groups. The median surgical time was 4.85 h [IQR: 4.23; 5.33] and 4.73 h [IQR: 4.07; 5.58] for the SoC and intervention group, respectively, *p*-value = 0.784. Aortic cross-clamping time was 83.00 min [IQR: 69.00; 105.00] and 72.50 min [IQR: 54.00; 95.00], *p*-value = 0.208 and CPB time was 106.00 [IQR: 90.00; 133.00] and 98.00 [IQR: 81.00; 136.00], *p*-value = 0.644, for SoC or intervention group, respectively. As for the type of primary filling of the cardiopulmonary bypass machine, it was the same composition for all patients: gelaspan^®^ (500 mL), HCO_3_ Na 1M (10 meq), plasmalyte 148^®^ (500 mL). Type of cardioplegia was the same for all patients, except for two where custodiol^®^ was used (one in each group). The cardioplegia solution for the other patients was prepared by the perfusionist team with cold blood from the patient, adding 60 mEq of Potassium and 2 g of Magnesium. Induction required 15 mL/kg and maintenance requires 10 mL/kg every 20 min. As for anesthesia, 56.7% received intravenous anesthesia (propofol) and 43.3% received a combination of intravenous and inhaled anesthesia (propofol and inhaled sevoflurane). Intravenous anesthesia was used in 55.1% of patients in the SoC and 58.33% in the intervention group without statistical differences between both groups (*p* = 0.838).

MAP during surgery was higher in the intervention group, 73 mmHg [IQR:70.30;76.10] and 77 [IQR: 71.96; 78.90] respectively, *p* = 0.008 but the percentage of time in which MAP was lower than 60 mmHg or 75% of baseline MAP as well as use of vasoactive drugs was similar in both groups (Table 2 Median lactic acid values before ECC were 8.6 mg/dL [IQR 7.1–11.3] and 8.55 [7.21–10.5], *p* = 0.646, for SoC and intervention group, respectively. During CPB, values increased to 10.25 mg/dL [8.35–13] and 10.3 [8.4–13.1], *p* value = 0.951 for SoC and intervention group respectively. Finally, the highest values were shown after CPB, also without significant differences between groups, 12.55 mg/dL [9.95–15.8] and 12.4 [9.5–16.5], *p* = 0.905, for SoC and intervention group, respectively. Forty patients needed transfusion during surgery (blood, platelets and/or plasma), twenty in each group.

Hemodynamic variables and fluid balance in the 24 h post-operative period for both groups are depicted in Table 3. There were not statistical differences in neither MAP (75.8 mmHg [IQR: 71; 79.4] vs. 76.15 mmHg [IQR: 73.2; 80.15] *p* = 0.32) nor MPP (67.15 [IQR 62.65; 70.4] vs. 66.7 [IQR: 64.15–71.2]; *p* = 0.375). The percentage of time in which MPP was less than 75% of baseline also similar in both groups (21.45 vs. 21.39% [*p* = 0.811]). As for vasoactive drugs, only norepinephrine use was more common in the intervention group (37.78 vs. 65.27%; *p* = 0.026).

As intervention included early furosemide initiation if high CVP when not contraindicated, an analysis of furosemide use, diuresis and fluid balance in first 24 h was also performed and no statistical differences were observed (Table 3).

### 3.3. CS-AKI Description

**Figure 2:**
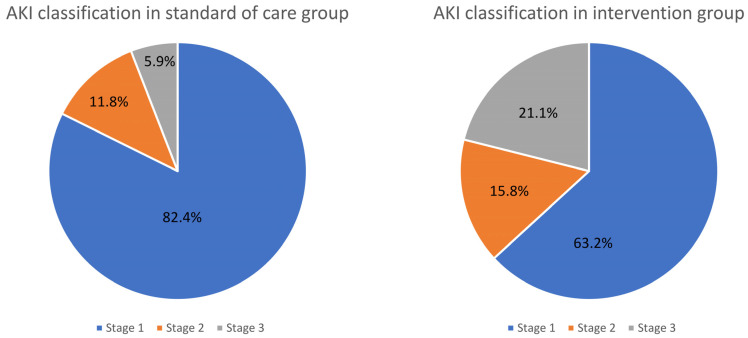
AKI stage distribution in intervention and standard of care groups.

There were no differences in sCr levels (1.15 mg/dL [IQR: 0.96; 1.42] vs. 1.3 mg/dL [IQR: 1.02; 1.54] *p* = 0.138) 24 h after surgery. Thirty-six patients (36.73%) suffered CS-AKI during the first week after surgery, seventeen in the SoC group and nineteen in the intervention group, without statistically significant differences (*p* = 0.404, for direct comparisons of proportions) and OR 1.19 (95%CI 0.51; 2.73), *p*-value = 0.65 from risk estimation with a logistic regression. The most common stage of AKI was stage 1 (72.22%) and two patients needed renal replacement therapy (one on each group, *p* = 1). The stages of AKI distribution are depicted in Figure 2.

All AKI diagnoses were performed during the first two days after cardiac surgery, except for one patient in the intervention group who suffered AKI five days after surgery. AKI progression at day seven after AKI started was assessed. Five out of thirty-six patients (13.89%) persisted with kidney dysfunction seven days after AKI started, two in the SoC group and three in the intervention group (*p* = 1).

### 3.4. MPP Deficit as a Risk Factor for CS-AKI

As the new target in our study compared to the standard protocol was maintaining MPP > 75% of basal value during first 24 h after surgery, we calculated the difference between basal MPP and mean MPP during first 24 h in order to ascertain whether a deficit of >25% in MPP was associated with AKI.

Seventeen patients (17.71%) presented an MPP deficit >25%, of which 8 (22.86%) suffered AKI, without statistically significant differences (*p* = 0.406). If we move this limit to a deficit of >20% we can observe that 29 patients (22.95%) had this deficit, of which 15 (42.86%) suffered AKI, almost reaching statistical significance (*p* = 0.064).

Other factors associated with AKI are depicted in Supplementary Table S1.

### 3.5. Patients Outcome

Median ICU length of stay and post-operative hospital length of stay were 4 days (IQR: 3–6) and 8 days (IQR: 6–11), respectively, without statistically significant differences between groups. Median ICU length of stay and post-operative hospital length of stay for SoC and intervention group were 3 days (IQR: 3–6) and 4 days (IQR: 3–6) for ICU length of stay, and 8 days (6–10) and 8 (6–12) for hospital length of stay, without statistical differences between groups. Three patients died during admission (3.06%), all of them in the intervention group. Patients died at the sixth, eleventh and ninety-fourth day after surgery and causes of death were bilateral pulmonary embolism, cardiac tamponade and refractory respiratory failure due to multiple complications, respectively. Ten patients needed a second surgery during hospital stay because of bleeding or cardiac tamponade (six in the SoC cohort and four in the intervention cohort). Two patients suffered a stroke in the post-operative period, one in each group: one patient suffered a bilateral pulmonary embolism in the intervention group, and one suffered a coronary artery spasm during surgery in the intervention group. In one patient in the SoC group, vegetation was found during surgery with final diagnosis of endocarditis.

Median creatinine and eGFR at discharge were 1.02 mg/dL (IQR: 0.81–1.23) and 68 mL/min (IQR: 57–90), respectively, without statistically significant differences between groups. The median time on vasoactive drugs was 2 days (IQR: 1–3) in the SoC group and 2 days (IQR 1–4) in the intervention group without differences (*p* = 0.481).

Transfusion description during surgery and first 24 h is depicted in Table 4. There were no differences in red blood cells, platelets nor plasma transfusion between groups.

## 4. Discussion

In this randomized clinical trial, patients at risk of CS-AKI based on Leicester score who followed an interventional strategy based on MPP deficit during surgery and the post-operative period did not have a lower incidence of AKI during the first seven days after surgery than those who received a SoC strategy.

Some observational studies have observed that deficit between a 20–25% in MPP in cardiac-surgery and septic patients is associated with AKI but significant limitations can be found regarding measurement of basal CVP, which can make the results inconsistent [6,8]. Saito S et al. [6] examined whether patients with CS-AKI had a greater difference between premorbid and within-ICU hemodynamic pressure-related parameters (including MPP) compared to those without. To note, CVP was estimated using inferior vena cava (IVC) parameters (diameter and collapse) derived from outpatient echocardiography examinations. As the echocardiography examination was not performed just before surgery, estimated CVP could have changed during waiting list period. It should be pointed out that hemodynamic parameters during surgery were not included in the study. Moving to studies in septic patients [7,8], basal parameters are even more difficult to estimate. CVP is assumed to be 6 mmHg in patients with a history of cardiovascular disease, and 2 mmHg in those without and basal arterial pressure is assumed to be 130/85 mmHg when no data are available. The present study has an accurate protocol of CVP and MAP measurement and we found that standard treatment at our institution maintained more than 75% of patients in MPP deficit <25%, without differences regarding to intervention strategy that included MPP targeted therapy. Moreover, neither 20 nor 25% of the deficit was statistically associated with AKI, although there was a trend regarding deficit >20% (*p* = 0.064). Our results suggest that good compliance with the gold standard (maintaining cardiac index > 2.2 L/min/m^2^ and MAP > 65 mmHg) is likely to maintain perfusion pressure.

Strategies that focus only on the post-operative period may fail since hemodynamic parameters during surgery need to be considered. More recently, Hu R et al. [13] aimed to explore the association between the difference in perfusion pressure during CPB compared to the baseline in the development of AKI in a retrospective study. Baseline MAP was estimated as diastolic blood pressure + 1/3 times pulse pressure difference. Baseline CVP was taken from the first post induction reading. Baseline MPP was derived from baseline MAP-baseline CVP. MPP during bypass was assumed, as we also did in our study, to equal MAP during bypass, as CVP falls to 0. In the study they found an association between AKI and cumulative number of median 3-min MPP values that were >20% below MPP baseline. Also, higher baseline CVP was independently associated with AKI.

There are noteworthy strengths of our trial. First, this is the first randomized controlled trial focused on target MPP in cardiac surgery, which allows an accurate measurement of baseline parameters which could not be possible in observational studies. It also allowed us to assess the safety of an intervention based on MPP. In our study, no differences in transfusion need, cardiovascular complications or mortality were found.

Our trial also has limitations. First, we acknowledge that even though algorithms were used in the postoperative period, they may be modified by the experience of the intensivist and there could be a bias, possibly accentuated by the non-blinded design. Second, due to the COVID-19 pandemic, the study failed to include the number of patients that was initially planned, with a consequent drop in statistical power.

In conclusion, in our study, individualized hemodynamic management based on MPP compared to standard treatment did not reduce the incidence of CS-AKI. A personalized MPP management is safe, but larger cohorts are needed to confirm these findings.

## Figures and Tables

**Table 1:** Cohort characteristics at baseline.

Characteristic	Total (*n* = 98)	Standard of Care (*n* = 49)	Intervention (*n* = 49)
Male, *n* (%)	81 (82.65)	40 (81.63)	41 (83.67)
Median age (IQR), yr	74.78 (67.24–78.78)	74.52 (67.24–78.78)	75.04 (67.46–78.37)
Median BMI (IQR), kg/m^2^	30 (27–33)	30.00 (27–32)	29.00 (27–33)
Smoking history, *n* (%)	64 (65.31)	31 (63.27)	33 (67.35)
Diabetes mellitus, *n* (%)	60 (61.22)	29 (59.18)	31 (63.27)
Hypertension, *n* (%)	84 (85.71)	40 (81.63)	44 (89.3)
Arteriopathy, *n* (%)	14 (14.29)	6 (12.24)	8 (16.33)
Median hemoglobin (IQR), g/L	133.5 (122–145)	134 (122–145)	133 (120–145)
Median eGFR (IQR), mL/min CKD stage 1 (%) CKD stage 2 (%) CKD stage 3 (%) CKD stage 4 (%)	54 (46–65) 5 (5.10) 23 (23.44) 65 (66.33) 5 (5.10)	55 (46–61) 1 (2.04) 11 (22.45) 35 (71.43) 2 (4.08)	53 (40–68) 4 (8.16) 12 (24.49) 30 (61.22) 3 (6.12)
Median Creatinine (IQR), mg/dL	1.27 (1.07–1.47)	1.26 (1.07–1.38)	1.32 (1.08–1.53)
Preserved ejection fraction (>50), *n* (%)	63 (64.29)	32 (65.31)	31 (63.27)
Urgent surgeries, *n* (%)	41 (41.84)	16 (32.65)	25 (51.02)
Type of surgery Valve substitution, *n* (%) Bypass graft, *n* (%) Combined procedure, *n*(%)	37 (37.76) 25 (25.55) 36 (36.73)	19 (38.78) 11 (23.45) 19 (38.78)	18 (36.73) 14 (28.57) 17 (34.69)
ACEi/ARB treatment, *n* (%)	67 (68.37)	32 (65.30)	35 (71.43)
Diuretic treatment, *n* (%)	61 (62.24)	26 (53.06)	35 (71.43)
Previous cardiac surgery, *n* (%)	5 (5.10)	3 (6.12)	2 (4.08)
Previous AKI, *n* (%)	7 (7.14)	2 (4.08)	5 (10.2)
Median Euroscore (IQR)	3.25 (2.26–5.30)	3 (2.03–4.54)	3.50 (2.53–6)
Median Leicester Score (IQR)	42.80 (35.30–51.80)	41 (33–49.95)	45 (35.80–53)
Median Charlson index (IQR)	6 (4–6)	6 (4–6)	6 (5–6)
Median MAP (IQR), mmHg	87 (80–95)	86 (80.5–93)	88 (80–96)
Median CVP (IQR), mmHg	10 (8–13)	11 (8–12)	10 (8–14)
Median MPP (IQR), mmHg	76 (69–83)	76 (70–82.5)	78 (69–87)

eGFR: estimated glomerular filtration rate; CKD: chronic kidney disease; ACEi/ARB: Angiotensin-converting enzyme inhibitors/*Angiotensin* II receptor blockers; MAP: mean arterial pressure; CVP: central venous pressure; MPP: mean perfusion pressure. AKI: acute kidney injury.

**Table 2:** Hemodynamic variables and fluid balance during surgery.

Characteristic	Total (*n* = 98)	Standard of Care (*n* = 49)	Intervention (*n* = 49)	*p* Value
Median MAP (IQR), mmHg	75.6 (70.80–78)	73 (70.3–76.1)	77.05 (71.96–78.9)	0.008
% time MAP < 75% baseline before CPB (IQR)	10.05 (2.6–22.1)	11.1 (2.6–21.1)	9 (2.8–24.13)	0.992
% time MAP < 60 mmHg before CPB (IQR)	3 (0–10)	3. 9 (0–14)	2.4 (0–5.5)	0.325
% time MAP < 75% baseline during CPB (IQR)	28.3 (15.85–48.4)	37.4 (13.7; 49.4)	23 (16–41.25)	0.325
% time MAP < 60 mmHg during CPB (IQR)	14 (8.5–24.3)	18.75 (8.65–32.65)	13 (8–18.8)	0.068
% time MAP < 75% baseline after CPB (IQR)	15 (5.3–33)	14.65 (4–35.1)	16 (8–29.5)	0.690
% time MAP < 60 mmHg after CPB (IQR)	5.40 (0–13.5)	5 (1–15)	5.4 (0–12)	0.864
Phenylephrine use, *n* (%)	88 (89.8)	43 (87.6)	45 (91.84)	0.740
Norepinephrine use, *n* (%)	78 (79.59)	35 (71.43)	43 (87.76)	0.078
Epinephrine use, *n* (%)	7 (7.14)	3 (6.12)	4 (8.16)	1
Efedrine use, *n* (%)	31 (31.63)	18 (36.73)	13 (26.53)	0.385
Dobutamine use, *n* (%)	51 (52.04)	26 (53.06)	25 (51.02)	1
Nitroglicerine use, *n* (%)	16 (16.33)	10 (20.41)	6 (12.24)	0.413
Furosemide use, *n* (%)	28 (28.57)	16 (36.65)	12 (24.49)	0.503
Diuresis (IQR), mL	417.5 (290–670)	410 (260–610)	470 (310–720)	0.366
Colloid use, *n* (%)	17 (17.53)	10 (20.83)	7 (14.29)	0.396
% of time Cardiac index < 2.2 (IQR) L/m^2^/min	12 (2.25–32)	10.6 (2.25–29.2]	18.1 (2–48.7)	0.384

MAP: mean arterial pressure; CPB: cardiopulmonary bypass.

**Table 3:** Hemodynamic variables and fluid balance in 24 h post-operative period.

Characteristic	Total (*n* = 98)	Standard of Care (*n* = 49)	Intervention (*n* = 49)	*p* Value
Median MAP (IQR), mmHg	76 (72.1–79.9)	75.8 (71–79.4)	76.15 (73.2–80.15)	0.320
Median MPP (IQR), mmHg	66.9 (63.8–70.85)	67.15 (62.65–70.4)	66.7 (64.15–71.2)	0.375
% time MPP < 75% baseline (IQR)	10.95 (1.55–32.75)	10 (2–32.9)	12.7 (1.20–28.80)	0.811
% time MAP 65 mmHg (IQR)	4.40 (1.1–12.5)	5.20 (1.3–16.9)	4.00 (1.1–11.1)	0.426
Norepinephrine use, *n* (%)	50 (51.02)	19 (37.78)	31 (63.27)	0.026
Efedrine use, *n* (%)	6 (6.12)	3 (6.12)	3 (6.12)	1
Dobutamine use, *n* (%)	47 (65.28)	23 (62.16)	24 (68.57)	0.597
Nitroglicerine use, *n* (%)	10 (10.20)	5 (10.2)	5 (10.2)	1
Nitroprussiate use *n* (%)	16 (16.33)	7 (14.29)	9 (18.37)	0.785
Furosemide use, *n* (%)	45 (45.92)	24 (48.98)	21 (42.86)	0.685
Diuresis (IQR), mL	1310 (1035–1625)	1285 (990–1565)	1320 (1060–1690)	0.236
Fluid balance (IQR), mL	225 (−716–1060)	331.00 (−384–1206)	−26.00 (−984–999)	0.154

MAP: mean arterial pressure; MPP: mean perfusion pressure.

**Table 4:** Transfusion need during surgery and first 24 h.

Characteristic	Total (*n* = 98)	Standard of Care (*n* = 49)	Intervention (*n* = 49)	*p* Value
During surgery, *n* (%)	RBC: 31 (31.63) Platelets: 12 (12.2) Plasma: 8 (8.2)	RBC: 14 (28.57) Platelets: 6 (12.24) Plasma: 4 (8.16)	RBC: 17 (34.69) Platelets: 6 (12.24) Plasma: 4 (8.16)	0.363
Cell-saver transfusion (IQR), mL	200 (0–305)	190 (0–295)	211.50 (0–315)	0.476
During first 24 h, *n* (%)	RBC: 19 (19.38) Platelets: 8 (8.16) Plasma: 15 (15.30)	RBC: 9 (18.37) Platelets: 4 (8.16) Plasma: 8 (16.33)	RBC: 10 (20.41) Platelets: 4 (8.16) Plasma: 7 (14.29)	0.494

RBC: red blood cells.

## Data Availability

The data that support the findings of this study are available on request from the corresponding author (E.P.).

## Supplementary Materials

The following supporting information can be downloaded at: https://www.mdpi.com/article/10.3390/jcm12247746/s1, Table S1: Variables associated with CSA-AKI.
