# Supplementary material for: Assessment of Individualized Mean Perfusion Pressure Targets for the Prevention of Cardiac Surgery-Associated Acute Kidney Injury—The PrevHemAKI Randomized Controlled Trial
Source: J Clin Med. 2023 Dec 18;12(24):7746. doi: 10.3390/jcm12247746 (PMC10743963; doi:10.3390/jcm12247746)
Supplement: Supplementary file 1 [file jcm-12-07746-s001.zip › jcm-2757177-supplementary.pdf]

Supplementary Table S1. Variables associated with CSA-AKI

	**No AKI patients**	**AKI patients**	**P value**
**Baseline characteristics**			
Median Creatinine (IQR), mg/dL	1.21 [0.99- 1.45]	1.36 [1.22- 1.48]	0.009
Diabetes mellitus, n (%)	43 (69.35%)	17 (47.22%)	0.034
**Surgical variables**			
% cardiac index <2.2L/min/m^2 (IQR)	8.73 (1.9-27)	18.9 (8-43.8)	0.046
ECC time (IQR), min	98.5 [76- 122]	128.5 [92.5- 147.5]	0.003
Surgical time (IQR), hours	4.65 [3.93-5.18]	4.94 [4.57-5.94]	0.003
Transfusion need, n (%)	22 (35.5)	18 (50)	0.02
Dobutamine after ECC, n, (%)	27 (43.55)	24 (66.67)	0.036
**First 24h**			
Transfusion need, n (%)	9 (14.52%)	13 (36.11%)	0.022
%Time MAP <65 mmHg (IQR)	3.6 [1- 9.10]	9 [1.15-21.07]	0.022

ECC: extracorporeal circulation; MAP: mean arterial pressure
